# Supplementary material for: ProteinShader: illustrative rendering of macromolecules
Source: BMC Struct Biol. 2009 Mar 30;9:19. doi: 10.1186/1472-6807-9-19 (PMC2672931; doi:10.1186/1472-6807-9-19)
Supplement: Additional file 1 — ProteinShader program without source code. This compressed file contains the complete ProteinShader program including associated libraries, but no source code. A README.txt file gives an overview of the ProteinShader distribution, and the index.html file in the help subdirectory has directions on getting started with the program as well as a set of tutorials. [file 1472-6807-9-19-S1.zip › ProteinShader-beta-0_9_4-binary/help/api/org/proteinshader/gui/listeners/menubar/package-summary.html]

org.proteinshader.gui.listeners.menubar (ProteinShader API)


|  |  |  |
| --- | --- | --- |
| **Overview** \| **Package** \| Class \| **Use** \| **Tree** \| **Deprecated** \| **Index** \| **Help** | |  |
| **PREV PACKAGE**   **NEXT PACKAGE** | **FRAMES**    **NO FRAMES**     **All Classes** |  |


---

## Package org.proteinshader.gui.listeners.menubar

Holds the listeners for the items used in the JMenus of the menu bar above
the canvas.

**See:**
  
          **Description**

| **Class Summary** | |
| --- | --- |
| **AboutBoxListenerFactory** | Creates the event listeners needed by the AboutBox. |
| **BackgroundMenuListenerFactory** | Creates the event listeners needed by the BackgoundMenu of the MainMenuBar. |
| **FileMenuListenerFactory** | Creates all event listeners needed by the FileMenu of the MainMenuBar. |
| **HelpMenuListenerFactory** | Creates all event listeners needed by the HelpMenu of the MainMenuBar. |
| **OrientationMenuListenerFactory** | Creates all event listeners needed by the OrientationMenu of the MainMenuBar. |
| **StyleMenuListenerFactory** | Creates all event listeners needed by the StyleMenu of the MainMenuBar. |
| **ToolsMenuListenerFactory** | Creates all event listeners needed by the ToolsMenu of the MainMenuBar. |
| **VisibilityMenuListenerFactory** | Creates all event listeners needed by the VisibilityMenu of the MainMenuBar. |

## Package org.proteinshader.gui.listeners.menubar Description

Holds the listeners for the items used in the JMenus of the menu bar above
the canvas.
